# A Metabolic Choreography of Maize Plants Treated with a Humic Substance-Based Biostimulant under Normal and Starved Conditions

**DOI:** 10.3390/metabo11060403

**Published:** 2021-06-20

**Authors:** Kgalaletso Othibeng, Lerato Nephali, Anza-Tshilidzi Ramabulana, Paul Steenkamp, Daniel Petras, Kyo Bin Kang, Hugo Opperman, Johan Huyser, Fidele Tugizimana

**Affiliations:** 1 Department of Biochemistry, University of Johannesburg, Auckland Park, Johannesburg 2006, South Africa; othibengkgalaletso3@gmail.com (K.O.); nephalipertunia20@gmail.com (L.N.); ramabulanaanza@gmail.com (A.-T.R.); psteenkamp@uj.ac.za (P.S.); 2 CMFI Cluster of Excellence, Interfaculty Institute of Microbiology and Medicine, University of Tubingen, Auf der Morgenstelle 28, 72076 Tübingen, Germany; functionalmetabolomics@gmail.com; 3 College of Pharmacy, Sookmyung Women’s University, Seoul 04310, Korea; kbkang@sookmyung.ac.kr; 4 International Research and Development Division, Omnia Group, Ltd., Bryanston, Johannesburg 2021, South Africa; Hugo.Opperman@omnia.co.za (H.O.); Johan.Huyser@omnia.co.za (J.H.)

**Keywords:** abiotic stresses, biostimulants, humic substances, metabolomics, molecular networking

## Abstract

Humic substance (HS)-based biostimulants show potentials as sustainable strategies for improved crop development and stress resilience. However, cellular and molecular mechanisms governing the agronomically observed effects of HS on plants remain enigmatic. Here, we report a global metabolic reprogramming of maize leaves induced by a humic biostimulant under normal and nutrient starvation conditions. This reconfiguration of the maize metabolism spanned chemical constellations, as revealed by molecular networking approaches. Plant growth and development under normal conditions were characterized by key differential metabolic changes such as increased levels of amino acids, oxylipins and the tricarboxylic acid (TCA) intermediate, isocitric acid. Furthermore, under starvation, the humic biostimulant significantly impacted pathways that are involved in stress-alleviating mechanisms such as redox homeostasis, strengthening of the plant cell wall, osmoregulation, energy production and membrane remodelling. Thus, this study reveals that the humic biostimulant induces a remodelling of inter-compartmental metabolic networks in maize, subsequently readjusting the plant physiology towards growth promotion and stress alleviation. Such insights contribute to ongoing efforts in elucidating modes of action of biostimulants, generating fundamental scientific knowledge that is necessary for development of the biostimulant industry, for sustainable food security.

## 1. Introduction

Currently, agriculture is facing a massive increase in demand due to twin pressures of an increasing population and environmental deterioration [1,2,3]. Hence, accurate and predictive metabolic models are imperative for designing a roadmap for the next generation of crops with high productivity and resilience to climate change, and devising agricultural strategies for sustainable crop production. Interrogating plant responses to environmental conditions, through the lenses of *omics* sciences, is disruptively enabling the decoding of the language of cells at molecular level. This advances the understanding of regulatory network rules and mechanistic events in the cellular and chemical space of the plant under consideration, which, in turn, provides greater impetus for the translation of fundamental knowledge to actionable programs in the field [4,5]. Thus, reported herein is an investigation of biostimulant-induced reconfigurations of maize metabolism towards growth enhancement and stress alleviation.

The incorporation of biostimulant strategies and programs in the agriculture industry holds promise to sustainably improve crop productivity. Currently, biostimulants, subdivided into microbial and non-microbial categories, are described as formulations that improve plant health and productivity as a resultant action induced by the novel, or emergent properties of the complex mixture, and not only from the presence of a plant growth regulator [6,7,8]. The biostimulant market is constantly on the economical rise due to the need to use formulations that promote sustainable soil health, and those that lead to crop improvement with respect to climate resilience and nutrition traits [3]. Emerging studies have demonstrated the effects of biostimulants on plant physiology and agronomic traits. For instance, the application of a biostimulant on tomato plants showed improved growth and fruit nutritional quality, as well as enhanced antioxidant machineries (e.g., elevation of ascorbic acid) under heat stress [9]. Another study by Paul et al. [10] investigated the action of protein hydrolysate-based biostimulants on tomato plants under drought, reporting biostimulant-induced changes in metabolic profiles and phenotypic traits of tomato plants. These changes included alterations in phytohormones and lipids, increases in biomass, stronger stomatal conductance, and enhanced antioxidant defence systems [10].

Despite ongoing efforts made in studying and understanding the effects of biostimulants on plants, the underlying biostimulant-induced changes (at molecular and cellular levels) for plant growth promotion and stress resilience remain an active research field. This knowledge gap hampers the novel formulation of biostimulants and the implementation of these products into agronomic practices [3]. Hence, in this work, metabolomics was applied to generate fundamental insights regarding the effects of humic substance (HS)-based biostimulants on maize metabolism under normal and nutrient-starved conditions. Metabolomics, a multidisciplinary *omics* science, provides a readout of the metabolome, which carries imprints of environmental and genetic factors. As such, one of the best descriptions of metabolism is the metabolic fluxes it generates, representing the integrated output of the molecular machinery and biochemical characteristics of a biological system [11,12]. Arguably, metabolomics is probably the most challenging and demanding of the *omics* sciences, due to the inherent complexity of the metabolome. However, metabolomics-generated insights are increasingly rendered possible as the field positions itself in the current innovative developments in analytical technologies, computational tools and integration of orthogonal biological approaches [12]. Thus, metabolomics offers unique opportunities in elucidating modes of action of biostimulants, at cellular and molecular levels, necessary insights for the biostimulant industry, and subsequently for a sustainable and improved cropping system.

## 2. Results and Discussion

As mentioned in the Introduction section, this study aimed at elucidating metabolic alterations that explain the effects of a non-microbial biostimulant, a humic substance (HS)-based formulation, on maize plants under normal and nutrient starvation, in greenhouse conditions. Experimental details are provided in Section 3. For semantic simplicity, the expressions humic substances, HS, humic biostimulant, HS-based biostimulant, and biostimulant will be used interchangeably to refer to the biostimulant formulation used in this study (a humic substance-based formulation, Section 3). Briefly, the study was designed to comprise four (4) different groups, namely, control 1 (starved and with no HS), control 2 (non-starved and no HS application), and HS-treated under starved and non-starved conditions (Table 1, Section 3). Prior to metabolomic analyses, the morphophysiological assessments were performed to evaluate the effects of the HS-based biostimulant on maize plants under normal and starved conditions. The HS-treated plants showed increased canopy cover, plant height, above ground dry biomass, improved nutrient uptake and nutrient leaf content under both normal and nutrient starved conditions (Figure S1). These morphophysiological traits observed in HS-treated plants can be associated with improved plant health, growth and nutrient stress alleviation. For metabolomics analyses, metabolites were extracted from leaves and analysed on liquid chromatography–mass spectrometry (LC-MS) analytical systems, with both untargeted and targeted approaches. Different methodologies and workflows were applied to mine and interpret the generated metabolomics data: these included molecular networking approaches, chemometrics methods, and metabolic pathway and network analyses (Section 3).

### 2.1. The Molecular Networking Approaches to Decode the Chemical Constellations of the Extracted Metabolome from Maize Plants

**Figure 1:**
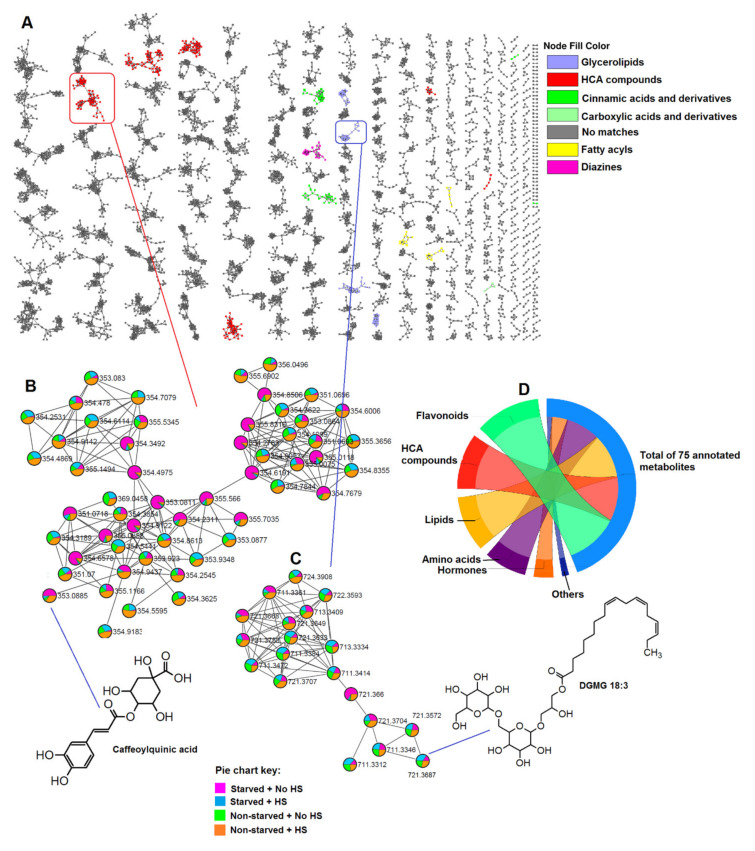
Metabolome coverage/atlas of leaves of nutrient-stressed and HS-treated maize plants and relative quantification of some of the matched classes. (**A**) Enhanced molecular network of the ESI-negative MS/MS spectra with a total of 7119 nodes obtained using MolNetEnhancer showing different molecular families/clusters of the pooled metabolites in the leaves of the maize plants under study. The coloured nodes represent classes of putatively annotated metabolites which were matched to GNPS libraries, whereas the grey nodes represent those unmatched to a library. Clusters of hydroxycinnamic acid (HCA) compounds (**B**) and glycerolipids (**C**) with pie charts showing differential changes in metabolite levels under different treatment conditions. (**D**) A chord plot/diagram showing classes of all the putatively annotated metabolites. GNPS link to spectral information of caffeoylquinic acid (*m*/*z* 353.08) is in the Supplementary File.

The spectra data (from untargeted analyses) were mined using computational tools such as feature-based molecular networking (FBMN) and MolNetEnhancer housed within the global natural product social (GNPS) molecular networking environment (Section 3). Using various algorithms, molecular networking provides a visual overview of all the ions of molecules that are detected and fragmented during an MS/MS experiment and the chemical relationship between them. This exploration of the collected ‘fragmentome’ enables the visualization of chemical similarity between annotated known metabolites and unknown molecules, thus expanding the coverage of the metabolome under consideration [13,14]. Moreover, in contrast to the conventional (also referred to as ‘classical’) molecular networking tool which relies solely on MS^2^ information for molecular network generation, FBMN improves upon this by also incorporating MS^1^ information such as retention time, ion mobility, and natural isotopic pattern. As a result, FBMN allows for spectral annotation, distinguishes isomers, as well as incorporates relative quantification information [15,16]. This method also offers the advantage of giving a more precise estimation of the relative ion intensity by making use of the LC-MS abundance of the features (i.e., peak area/height), as opposed to classical MN which makes use of the sum/total precursor count or spectral count [15]. The MolNetEnhancer workflow, on the other hand, improves the chemical insight obtained from a dataset by combining outputs from multiple independent computational tools such as molecular networking, MS2LDA (MS2 latent Dirichlet allocation), as well as the Network Annotation Propagation (NAP) in silico annotation tools and thus allowing for enhanced metabolomics data annotation [15]. In this study, the metabolome covered included unknown classes which had no matches and known or putatively annotated classes, namely, glycerolipids, hydroxycinnamic acid (HCA) compounds, cinnamic acids and derivatives, carboxylic acids and derivatives, fatty acyls and diazines (Figure 1A), and, as detailed in the methodology (experimental, Section 3), more confirmatory scrutiny was performed to validate the metabolite annotations.

Thus, FBMN and MolNetEnhancer both aided in the putative annotation of some of the metabolites in the extracted maize leaves metabolome. Each node represents a single chemical entity, e.g., caffeoylquinic acid (*m*/*z* 353.0885, Figure 1B) and DGMG 18:3 (gingerglycolipid A-*m*/*z* 721.3687, Figure 1C), which can be connected to other structurally similar chemical entities (nodes) by edges in a cluster, molecular family. The putatively annotated metabolites/nodes can then, in turn, be used for the identification of other nodes in the same molecular family by means of the extrapolation of loss or gain of certain chemical groups [17]. Furthermore, the molecular networking computation also provided a quantitative description of the measured metabolome, pointing to the differential distribution of ions belonging to different classes, as reflected on the pie charts in the clusters of HCA compounds and glycerolipds, showing the effect of HS on the maize plants under normal (well-fed) and stress (starved) conditions (Figure 1B,C). This is further discussed in the subsequent sections. The extracted and annotated maize metabolome comprised different classes of metabolites, as infographically shown in Figure 1D, suggesting that the metabolic changes (in maize plants) induced by treatments span a wide spectrum of both primary and secondary metabolic phenomenology.

### 2.2. HS-Biostimulant Alters Maize Primary and Secondary Metabolism towards Growth Promotion

**Figure 2:**
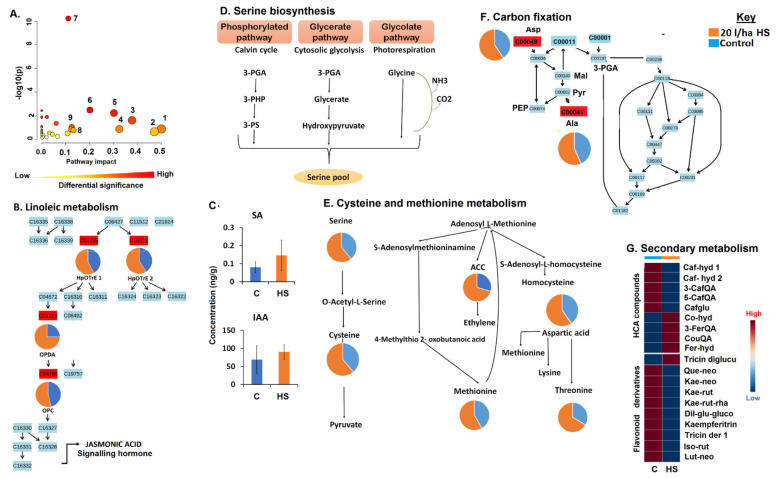
A summary of metabolic pathway analysis generated using MetPA, pathway mapping and relative quantification of some altered amino acid, hormones, oxylipins and phenolic compounds. (**A**) The graph displaying the ‘metabolome view’ containing all the mapped pathways arranged by *p*-values on the *y*-axis and the pathway impact (differential significance) on the *x*-axis. (1) Isoquinoline alkaloid biosynthesis, (2) phenylalanine metabolism, (3) alpha-linolenic acid metabolism, (4) tryptophan metabolism, (5) glycine, serine and threonine metabolism, (6) cysteine and methionine metabolism, (7) aminoacyl-tRNA biosynthesis, (8) stilbenoid, diarylheptanoid and gingerol biosynthesis (9), refer to Table S1. (**B**) Linoleic metabolism. (**C**) Absolute quantification of selected hormones. (**D**) Serine biosynthesis. (**E**) Cysteine and methionine metabolism. (**F**) Carbon fixation in photosynthetic organisms. (**G**) Secondary metabolism, relative quantification of selected phenolics. Abbreviation: 3-PGA, 3-phosphoglyceric acid; 3-PHP, 3-phosphohydroxypyruvate; 3-PS, 3-phosphoserine; Mal, malate; Pyr, pyruvate; PEP, phosphoenolpyruvate. Other abbreviations are found in Table S2.

The application of HS-based biostimulant on maize plants (under normal conditions) induced coordinated changes in the maize chemical space (Figure 1), significantly impacting pathways for primary and secondary metabolism. Some of these metabolic pathways include alpha-linolenic acid metabolism, amino acid-related pathways (such as tryptophan metabolism, glycine, serine and threonine metabolism and cysteine and methionine metabolism), and secondary metabolism pathways such as phenylpropanoid pathway and flavonoid metabolism (Figure 2A; Table S1). Maize plants treated with the humic biostimulant showed increased levels of oxylipins such as oxo-phytodienoic acid (OPDA), hydroperoxy-octadecatrienoic acid (HpOTrE) 1, hydroperoxy-octadecatrienoic acid (HpOTrE) 2 and oxo-(pentenyl)cyclopentaneoctanoic acid (OPC), components of alpha-linolenic acid metabolism (Figure 2B). Although the mechanistic roles of these individual oxylipins are still poorly understood, some of the general functions of oxylipins in plants include modifications of chloroplast function, plant senescence, stomatal conductance, and antifungal and antibacterial activities [18]. Furthermore, the oxylipin pathway leads to the generation of the phytohormone, jasmonic acid. Moreover, other signalling metabolites such as indole acetic acid (IAA) and salicylic acid (SA) were found increased in maize plants treated with HS compared to the control (Figure 2C). These phytohormones are regulatorily involved in various biochemical and physiological processes in plants, such as seed germination, seedling growth, stomatal aperture, respiration, and in interactions with the environment [19,20]. Thus, the measured changes in lipids and hormonal (signalling) networks in maize plants (Figure 2B,C) suggest that the HS biostimulants remodel maize metabolism towards growth promotion via the activation and enhancement of physiological events for improved plant development and the potentiation of defences [21,22,23].

Furthermore, other metabolic remodelling induced by the HS treatment on maize plants under normal conditions included a general increase in the levels of amino acids (Figure 2E,F). Amino acids play indispensable roles in metabolic pathways governing the plant growth and development processes. In this study, HS application increased the content of alanine (Ala) and aspartic acid (Asp), amino acids which are involved in the carbon assimilation/fixation pathway (Figure 2F), one of the essential processes in growth promotion. Plants do not only harvest atmospheric carbon dioxide for the production of photosynthates, they also utilize the internal carbon pool [24,25]. Thus, it can be postulated that increases in Ala and Asp levels contribute to an increased pool of the internal carbon, which could be used in photosynthetic reactions, thus supporting growth promotion. Asp also plays an important role in maintaining plant growth by serving as a substrate/precursor for the biosynthesis of four essential amino acids, namely, Thr, Lys, Ile and Met via the Asp-family pathway (Figure 2E) [26]. The increased levels of Thr and Met could be the result of the upregulation of the Asp-family pathway in HS-treated plants (Figure 2E). Correspondingly, the study of Vaccaro et al. [27] showed a significantly higher accumulation of Thr in the leaves of seedlings grown with HS in comparison to those observed in control plants. Met is also involved in a wide range of functions in plant growth and development; for example, it provides a required supply of sulphur and nitrogen to plants [28]. Thus, in this study, it can be postulated that the HS-induced increased level of Met was also translated into the measured increase in sulphur and nitrogen contents (Figure S1A), a growth promotion mechanism. Moreover, Met is also known to maintain the structure of proteins required for cell differentiation and division [28].

Other changes in amino acid levels included an increase in Ser levels in HS-treated maize plants, under normal conditions (Figure 2E). Ser is synthesized through three routes: (i) the glycolate pathway (photorespiration); (ii) glycerate pathway (cytosolic glycolysis); and (iii) phosphorylated pathway (Calvin cycle) (Figure 2D). Thus, our results suggest that the application of HS may have impacted these pathways, leading to the accumulation of Ser (Figure 2E). Apart from its proteinogenic roles, Ser takes part in the biosynthesis of several biomolecules required for cell proliferation, including amino acids, nitrogenous bases, phospholipids, and sphingolipids. Furthermore, it also plays an indispensable role in signalling mechanisms, as one of the three amino acids that are phosphorylated by kinases [29]. Ser is also involved in another significantly impacted pathway: Gly, Ser and Thr metabolism (Figure 2A; Table S1), which plays an important role in plant photorespiration [26]. The accumulation of amino acids in HS-treated plants (Figure 2E,F and Figure S2A) also suggests an increased pool of substrates for protein synthesis, which is positively associated with increased plant biomass [30]. Agreeably, these metabolic measurements were translated into the maize phenotype, because HS-treated plants showed higher plant biomass, an HS-enhanced growth and development (Figure S1C).

The application of the HS-based biostimulant on maize plants under normal conditions also impacted the secondary metabolism, as revealed by molecular networking approaches (Figure 1) and metabolic pathway analysis (Figure 2A,G and Table S1). In this study, under normal conditions, most flavonoids such as quercetin, luteolin neohesperidoside, kaempferol and isorhamnetin rutinoside levels were decreased in plants treated with HS compared to non-treated plants (Figure 2G). Moreover, the application of HS showed a differential response of HCA compounds, namely, chlorogenic acids and cinnamoyl hydroxycitric acid esters (Figure 2G). Primary and secondary metabolisms are involved in the use of the available photosynthetic assimilates, leading to trade-offs of the carbon allocation. In nutrient-rich environments, large amounts of carbohydrates are allocated to primary metabolism (protein synthesis), while secondary metabolism (phenolics production) is limited [31,32]. The latter could be a possible reason for the observed reduction in flavonoid contents in HS-treated plants compared to control plants, under normal conditions.

Furthermore, the decreased levels of some HCA compounds (3- and 5-caffeoylquinic acid, caffeoyl hydroxycitric acids and caffeoylglutarate; Figure 2G) may suggest that the phenylpropanoid pathway was not favoured in HS-treated plants under normal physiological conditions, regardless of the accumulation of the precursors of this pathway, of Phe and Tyr in HS-treated plants vs. control (Figure S2A). This result further supports the above-mentioned hypothesis that the carbon from these amino acids is mainly directed towards the primary metabolism, thereby prioritizing plant growth. With the phenylpropanoid pathway not being stimulated, this may have affected the downstream pathways such as flavonoid metabolism; thus, a general decrease in flavonoids levels in HS-treated plants (Figure 2G). However, some phenolic compounds such as tricin diglucuronide, 3-feruloylquinic acid, coumaroylquinic acid and coumaroyl hydroxycitric acid were increased under HS treatment (Figure 2G). This points to a dynamic and complex network of phenolic compounds, reconfigured by biostimulant treatment for the enhancement of growth and development of maize plants, under normal conditions [33]. These HS biostimulant-induced metabolic alterations (accumulation of lipids, hormones and amino acids and differentially changed phenolic compounds) under normal conditions (Figure 2) were synchronously translated into agronomic traits: the maize plants treated with the humic substances showed increased canopy cover, plant height, plant diameter, above ground dry biomass and chlorophyll content (Figure S1C), and enhanced plant growth mediated by HS-based biostimulant application.

### 2.3. HS-Biostimulant Alleviates Nutrient Starvation in Maize Plants: Underlying Metabolic Reprogramming

**Figure 3:**
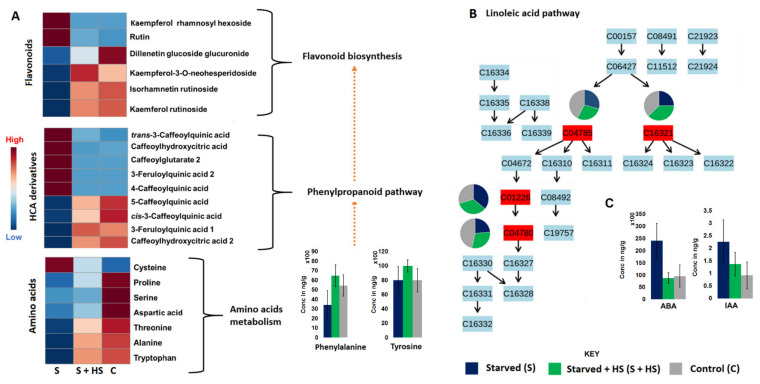
Relative quantification and pathway mapping of annotated metabolites under nutrient starvation. (**A**) Heatmaps and bar graphs showing the relative (and absolute) quantification of amino acids, HCA derivatives and flavonoids. (**B**) Pathway mapping of annotated oxylipins and their differential distribution in control, HS-treated and untreated under nutrient starvation. (**C**) Bar graphs showing the absolute quantification of some hormones, ABA and IAA; *p*-values are reported in Table S3.

The HS-biostimulant-induced global metabolic reprogramming under nutrient starvation spanned a wide range of metabolic classes such as flavonoids, HCA compounds, lipids, amino acids and hormones (Figure 1). Chemometrically, in principal component analysis (PCA) scores (Figure S2B), the nutrient-starved group which was treated with HS (S + HS) clustered closely to the non-starved group, suggesting similar metabolic profiles in the two groups. Correspondingly, relative quantification analysis also revealed that the amino acid and phenolic compound (HCA derivatives and flavonoids) profiles of the starved plants treated with HS (S + HS) are similar to the profiles of the non-starved plants (Figure 3A). Amino acids were significantly reduced due to nutrient starvation in non-HS-treated plants (Figure 3A), suggesting an increased degradation of amino acids as an alternative mechanism to compensate for limited nitrogen (N) and/or carbon (C) supply. Enhanced amino acid degradation is usually observed in plants suffering from C deficiency [34]. However, the application of HS to starved plants showed an increase in these amino acids compared to non-treated starved plants (Figure 3A). This could mean that HS either directly supplies the plants with C and N or it triggers other mechanisms which efficiently provide the plant with sufficient C and N. Several studies have shown that the application of HS enhances the acquisition and mobilization of nutrients such as N (amongst others). N is known as the most essential nutrient in plants, because its metabolism is the basis of biological molecules such as amino acids, proteins, nucleotides and enzyme synthesis [35,36,37]. The increase in amino acids observed in starved HS-treated plants compared to the untreated starved plants (Figure 3A) can thus be correlated with the increased absorption of N (Figure S1).

With regard to the HCA compounds and flavonoids, metabolites which were increased in non-treated starved plants (e.g., rutin, kaempferol rhamnosyl hexoside, trans-3-caffeoylquinic acids, 3-feruloylquinic acid 1, caffeoylhydroxycitric acid, etc.) were decreased in HS-treated starved plants (Figure 3A). In contrast, phenolic compounds that were decreased in non-treated starved plants (e.g., kaempferol rutinoside, isorhamnetin rutinoside, cis-3-caffeoylquinic acid, etc.) were increased in HS-treated starved plants (Figure 3A). The application of HS also showed a differential response of oxylipins such as OPDA, HpOTrE 1, HpOTrE 2 and OPC (Figure 3B). Oxylipins have been shown to be involved in stress signal transduction, the regulation of stress-related gene expression, and interaction with hormonal signalling pathways [38]. The growth and stress hormones, IAA and ABA (abscisic acid), respectively, were decreased by the application of HS under nutrient starvation (Figure 3C), suggesting homeostasis (towards normal condition). Generally, under abiotic stress conditions, plants biosynthesize higher levels of ABA, which induce stomatal closure and inhibit the growth and development of plants [39,40]. The level of IAA was increased under nutrient starvation in non-treated maize plants (Figure 3C), which correlated with previous studies [41].

Overall, these metabolic alterations suggest that the application of HS under nutrient starvation induces metabolic readjustments to alleviate the negative effect of starvation in plants. It can then be postulated that HS-based biostimulant treatment led to a rewiring of the maize metabolism for the efficient acquisition and use of resources under limited supplies of nutrients. This HS-induced metabolic remodelling towards stress alleviation correlates to the observed in-plant nutrient profiles; the uptake of macronutrients such as K, N, Ca, Mg, P and S and micronutrients such as Na, Fe, Zn, Mn, B and Cu was higher in starved plants that were treated with HS biostimulant compared to non-treated plants (Figure S1A). Moreover, the nutrient leaf analysis showed that the leaves of HS-treated starved plants contained higher levels of nutrients compared to non-treated starved plants (Figure S1B). Furthermore, these metabolic changes (and nutrient profiles) were translated into phenotypically observable agronomic traits such as improved plant height, above ground dry biomass, and canopy cover (Figure S1C).

**Figure 4:**
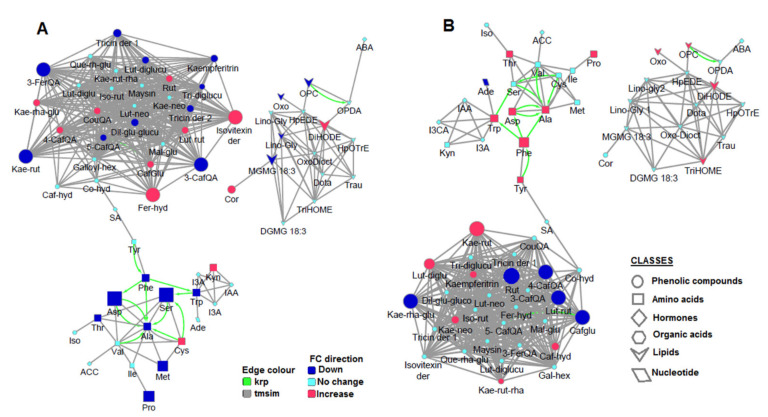
MetaMapp metabolite network visualization depicting the effects of nutrient starvation on (**A**) non-treated plants and (**B**) HS-treated plants. Red nodes indicate increased metabolites, whereas the blue indicates a decrease. Node size indicates the magnitude of fold-change. Compounds are connected by KEGG reaction pair (krp, green line), and chemical similarity (grey line).

To distinctively map and globally visualize the metabolomic data, a metabolic network analysis was performed using MetaMapp. This web-based tool is able to map all detected metabolites into network graphs using the KEGG reactant pair (krp) database and Tanimoto chemical similarity between PubChem substructure fingerprints, thus generating an overview of the metabolic regulation under specified conditions [42]. The chemical similarity feature was implemented on the foundation that biochemistry is described as the inter-conversion of chemically similar entities. This information can thus assist in the prediction of the enzymatic transformation networks between the biochemical domains [43]. As infographically depicted on the metabolic networks (Figure 4), there are three main metabolic clusters, namely, phenolics (indicated by the circles), lipids (arrows), and amino acids (squares), which are mainly interconnected based on their chemical similarity (grey edges). Hormones (diamonds) such as indoles (e.g., IAA) and ABA are structurally interlinked with amino acids and lipids, respectively. Thus, the correlation network computed comprised structural similarity complemented by krp interactions to avoid the misclustering of some obviously biologically related compounds and to reveal the biochemical reaction networks [44,45]. The krp interactions (highlighted in green) are shown between amino acids (Ala–Ser, Ala–Asp, Ala–Cys, Ala–Val, Ala–Phe, Phe–Tyr, Ser–Trp, Cys–Ser) and between oxylipins OPC and OPDA (Figure 4). The biochemical reaction network amongst the amino acids highlights Ala as a metabolite hub of the network, with many krp edges connecting to the Ala node (Figure 4). This point to the tight regulation of the amino acid metabolism and may warrant a closer look into the potential roles of Ala as a regulator. Ala metabolism has been shown to be tightly linked to carbon and nitrogen metabolism, the TCA cycle and sugar metabolism [46].

Furthermore, MetaMapp analysis utilizes statistical information such as the *p*-value and fold-changes [43]. Thus, the generated metabolic networks revealed significantly altered metabolites in HS-treated (Figure 4B) and non-treated (Figure 4A) plants under nutrient starvation (illustrated by node attributes such as size and colour). Ala was decreased in non-treated plants in response to nutrient starvation, and the other amino acids which are connected to Ala were also decreased (Figure 4A). However, in HS-treated starved plants, Ala was increased while its interconnections were either increased or unchanged (Figure 4B). Moreover, a study by Ishihara et al. [30] showed that the enrichment in free Ala was the best choice to correct enrichment in alanine residues in protein and determine the accurate rate of protein synthesis plants. This further supports the functional role of Ala as a potent regulator of amino acid metabolism. Furthermore, the application of these metabolic network maps allowed for the detection of metabolites which were significantly altered by HS application under starvation. For instance, observing the phenolics cluster, compounds such as kaempferol rutinoside, rutin, luteolin rutinoside, and caffeoylglutarate were significantly changed compared to other compounds which showed no significant changes (Figure 4B; see Table S3 for *p*-values). This suggests that one of the mechanisms employed by HS in stress alleviation involves upregulation/downregulation of specific phenolic compounds—A complex and dynamic network of phenolic compounds, as also reflected in Figure 2G. The computed metabolic network (Figure 4) points to possible regulatory events underlying the HS-induced metabolic reconfiguration in maize plants towards growth enhancement and the alleviation of nutrient starvation.

**Figure 5:**
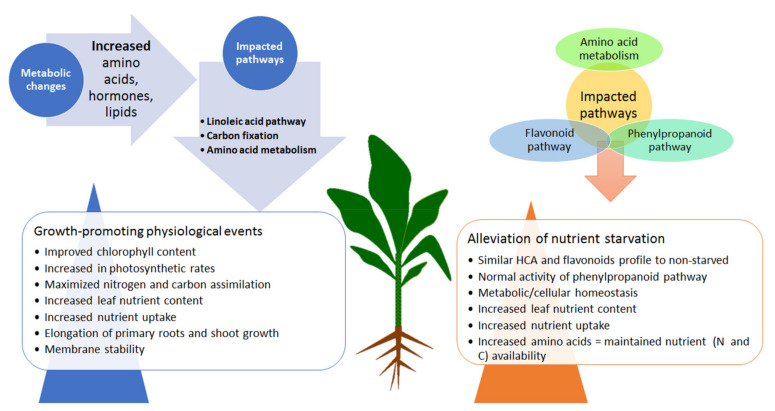
A contextual summary of postulated mechanisms elucidated from this study. The left side of the plant highlights the changes in metabolites involved in the key impacted pathways, leading to growth-promoting physiological events under HS treatment in non-starved conditions. On the right side of the plants are the biochemical alterations in the levels of metabolites spanning the impacted pathways identified in the HS-treated, starved plants, which were associated with the HS-enhanced alleviation of nutrient starvation.

Thus, a mechanistic model emerging from the present study provides key fundamental insights describing a hypothetical (metabolic) framework underlying the effects of HS-based biostimulants on maize plants, under normal and nutrient-starved conditions (Figure 5). Metabolic reconfigurations related to the HS biostimulant-induced growth promotion involves differential alterations in the levels of amino acids, phenolics and lipids, which are translated into physiological events such as (i) membrane remodelling, (ii) improved chlorophyll content and photosynthesis rates, (iii) improved N and C assimilation, (iv) elongation of roots and shoots, and (v) increased nutrient uptake and assimilation (Figure 5). The main HS-mediated mechanisms involved in nutrient stress alleviation elucidated in this study include (i) metabolic/cellular homeostasis, (ii) low-cost machineries in response to starvation, and (iii) increased nutrient uptake and assimilation.

## 3. Materials and Methods

The maize (*Zea mays*) plants, PAN 3Q-240, were cultivated in 10 L pots filled with 17 kg of sandy soil (pH of 4.6), organic carbon of 0.22% m/m, bulk density of 1495 kg·m^−3^ and organic matter of 0.38% m/m) in a greenhouse on a rotating table, at Omnia facilities in Sasolburg, Free-State, South Africa. The study was experimentally designed to comprise different treatments or groups (Table 1), i.e., plants with no HS-treatment and starved (Control 1), plants with no HS-application and no starvation (Control 2), and two HS-treated groups (with and without starvation). Each pot was considered as a biological replicate and contained five plants at the harvesting time. Five biological replicates (i.e., five pots) per treatment (group) were harvested. Immediately after emergence, well-fed plants were given NUTRIGRO^TM^ and NUTRIPLEX™ at 1 g/L of water of each product (100%) and nutrient-starved plants were given 0.4 g/L of water of each product (40%). At the 4-leaf stage, 20 L/ha humic substance-based biostimulant formulation (Omnia Group Ltd., Bryanston, South Africa), was applied to treatment groups (Table 1). The detailed descriptions and preparation of this HS-based formulation are not disclosed, because these biostimulant products are Omnia trade-marked and still undergoing commercialization processes. Harvesting of the plant materials was performed 3-days after the application of humic substances. The leaves were harvested and immediately shock-frozen in liquid nitrogen to quench all metabolic reactions [47,48]. The frozen plant leaf tissues were stored at −20 °C, pending metabolite extractions.

### 3.1. Metabolite Extraction

For metabolite extraction, the harvested leaf samples were crushed (to a fine powder) using liquid nitrogen in a mortar. Two grams (2 g) of the crushed leaves were weighed, dissolved in 20 mL (1:10 *m*/*v*) of 80% analytical grade cold methanol, and then subjected to homogenisation using a probe, Ultra-Turrax homogenizer, at 100% intensity for 2 min. Following homogenisation, the mixtures were sonicated for 30 s at 55% power using a probe sonicator (Bandelin Sonopuls, Berlin, Germany) and the crude extracts were centrifuged at 5100 rpm for 20 min. The supernatants were evaporated under vacuum to approximately 1 mL using a Büchi Rotavapor R-200 (Heidolph Laborota, Schwabach, Germany) at 55 °C, transferred to 2 mL Eppendorf microcentrifuge tubes, and then dried to completion using a speed vacuum concentrator (Eppendorf, Merck, Johannesburg, South Africa) set at 45 °C. The dried residues were then resuspended with 500 µL of LC-MS-grade methanol: milliQ water (1:1, *v*/*v*) and filtered into HPLC vials (Shimadzu, South Africa). The quality controls (QCs), consisting of pooled equivalent volumes from the control and treatment groups, were prepared. The filtered samples were stored at 4 °C until analysis.

### 3.2. Data Acquisition Using Liquid Chromatography–Mass Spectrometry Systems

An ultra-high-performance liquid chromatography (UHPLC) system coupled to a high-definition quadrupole time-of-flight MS instrument (Waters Corporation, Manchester, UK) was used to analyse the aqueous-methanol extracts, for the nontargeted approach. The samples were chromatographically separated prior to MS analysis on the UHPLC system fitted with an Acquity HSS T3 C18 column (Waters, Milford, USA, 1.7 μm, 150 mm × 2.1 mm) at a flow rate of 0.4 mL/min. A sample volume of 2 µL was injected, and the column was housed in a column oven thermostated at 60 °C. The binary solvent system comprised solvents A (0.1% aqueous formic acid in Milli-Q water) and B (0.1% formic acid in acetonitrile). The initial conditions (98% solvent A and 2% solvent B) were maintained for 1 min. The conditions were then gradually changed to 30% solvent A and 70% solvent B at 14 min, followed by a change at 15 min to 5% solvent A and 95% solvent B, which were maintained for 2 min and then changed to the initial conditions at 18 min. The analytical column was allowed to calibrate for 2 min before the next injection. The total chromatographic run time was 20 min.

The chromatographic effluent was further analysed as follows: a SYNAPT G1 high-definition mass spectrometer, equipped with electrospray ionization (ESI) source, was used for untargeted analysis. The MS detector was set to acquire centroid data in both positive and negative ionisation modes. The MS conditions used were as follows: the source temperature was set at 120 °C, desolvation temperature at 450 °C, capillary voltage 2.5 kV, sampling and extraction cones at 30 V and 4 V, respectively, cone gas flow at 50 L h^−1^, desolvation gas flow at 550 L/h, and a mass scan range of 50–1200 Da with a scan time of 0.1 s and an inter-scan delay of 0.02 s. Analysis of each sample was performed in triplicates. Online mass correction was conducted using a lock spray source: leucine encephalin (50 pg/mL), [M + H]^+^ = 556.766, and [M − H]^−^ = 554.2615, to ensure high mass accuracy (1–3 mDa) of analytes. For downstream structural elucidation, the MS analyses were set to result in both unfragmented and fragmented experiments through collision-induced dissociation (MS^E^), where the fragmentation patterns were obtained by alternating the collision energy from 10 to 50 eV. For targeted analysis, a triple quadrupole mass spectrometry platform, LCMS-8050 (Shimadzu, Kyoto, Japan), equipped with an ESI source and ultra-fast liquid chromatography (UFLC) as the front-end, was utilized. A multiple reaction monitoring (MRM) method was used for absolute quantification of the targeted metabolites (amino acid and hormones) (Table S4): descriptions of the LC and MS parameters are detailed in Nephali et al. [49].

### 3.3. Data Mining: Data Processing and Multivariate Data Exploration

The UHPLC-qTOF-MS raw data were processed using MassLynx XS™ software’s MarkerLynx application (Waters, Manchester, UK). This application makes use of the patented *Apex*Track algorithm [50] to perform accurate peak detection and alignment and results in a data matrix of retention time (Rt) *m*/*z* variable pairs, with *m*/*z* peak intensity for each sample. The following parameters were used for data processing: retention time (Rt) range of 1–17 min, a 100–1100 Da mass range, intensity threshold of 50, mass tolerance of 0.05 Da, and an Rt window of 0.2 min for both polarities. Normalization was then performed by using total ion intensities of each defined peak; prior to calculating intensities, the software performs patented modified Savitzky–Golay smoothing and integration. Only data matrices with noise levels below 50% (MarkerLynx metrics) were used for downstream data analysis strategies. The data matrices generated from MassLynx were exported into the SIMCA-15.0 software (Umetrics Corporation, Umea, Sweden) for statistical modelling. Some of the computed chemometrics models were included principal component analysis (PCA). The latter is an unsupervised method that aims at data dimensionality reduction and generates a model that reveals clusters, trends, and similarities between treatment groups [12]. Supervised, orthogonal partial least squares-discriminant analysis (OPLS-DA) models were also computed for (binary) sample classification and generating the descriptive statistics. MetaboAnalyst (version 5.0) was used for further statistical analyses where necessary.

Before building the chemometrics models (e.g., PCA or (O)PLS-DA), data pre-treatment (e.g., pareto scaling) was applied to normalize the variances and correct heteroscedasticity [51,52]. A nonlinear iterative partial least squares algorithm (in-built within SIMCA software) was used to handle the missing values, with a correction factor of 3.0 and a default threshold of 50%. A sevenfold cross-validation (CV) method was applied as a tuning procedure in generating the models, and only the components positively contributing to the prediction ability of the model (*R1* significant components) were considered. Furthermore, different metrics and tests were used for model validation, which included an evaluation of explained and predicted variation (cumulative R^2^ and Q^2^), the analysis of variance testing of cross-validated predictive residuals (CV-ANOVA, *p*-value < 0.05 as a cut-off), the receiver operating characteristic (ROC) curves, response permutation tests (with *n* = 100) and predictive testing. Thus, to ensure reliable results, only thoroughly validated and (preferably) parsimonious models were considered in this study. Quantitative analysis (i.e., generation of comparative bar graphs, heatmaps and pie charts) was performed using average integrated peak areas and concentrations for untargeted and targeted metabolites, respectively.

### 3.4. Molecular Networking

All the raw vendor (i.e., Waters) format MS/MS data were first converted to ‘analysis base file’ (ABF) format using the Reifys Abf converter software (https://www.reifycs.com/AbfConverter/, accessed on 21 April 2021) and then uploaded into the Mass Spectrometry-Data Independent AnaLysis (MS-DIAL) software. The MS-DIAL data-processing program makes use of a deconvolution algorithm to perform mass spectral deconvolution of data-independent acquisition (DIA) data, thus making it applicable for the extensive untargeted metabolomics analysis of both DIA and data-dependent acquisition (DDA) centroid datasets [53]. The data were processed using the following parameters: mass accuracy (MS1 and MS2 tolerance) of 0.05 Da, minimum peak height of 50 amplitude and mass slice width of 0.1 Da for peak detection, a 0.5 sigma window value and a 0 MS/MS abundance cut-off for data deconvolution; a retention time tolerance of 0.05 min was used under alignment parameter settings with one of the QC samples used as a reference file for alignment. Following data-processing with MS-DIAL, the resultant GNPS export files, i.e., GnpsMgf and GnpsTable (feature quantification table) were then uploaded into the GNPS environment (https://gnps.ucsd.edu/, accessed on 28 April 2021) using the WinSCP server for molecular networking.

A feature-based molecular network (FBMN) was generated for both the negative and positive mode data by uploading the respective feature quantification table, MGF file and a metadata file describing the properties of the sample file (i.e., treatment, days, plant condition, HS concentration and stress level). The MS/MS (fragmentation) spectra were clustered using the MS-Cluster algorithm with a precursor ion mass tolerance of 0.05 Da and fragment ion mass tolerance of 0.05 Da to create the consensus spectra. A network was generated where the lines/edges connecting the nodes were filtered to have a cosine score above 0.7 and a minimum of 4 corresponding fragment ions. This approach builds on the assumption that molecules which are structurally related give rise to similar fragmentation patterns when subjected to MS^2^ fragmentation, for example, collision-induced dissociation (CID), thus allowing for molecular networks to be created [14,54]. The MN spectra were then searched against the spectral libraries housed in GNPS where the same parameters (i.e., cosine score > 0.7 and min-matched fragments of 4) were used for metabolite annotation. The resultant molecular network data were first enhanced with the MolNetEnhancer to improve the chemical structural annotations acquired before they were visualized using the Cytoscape network visualization tool/software (version 3.8.2), where the nodes and edges were labelled and coloured. For the FBMN networks, the nodes were labelled with the precursor mass (*m*/*z*) and coloured by means of pie charts based on the differential changes in the metabolite levels under different treatment conditions. The MolNetEnhancer networks, on the other hand, were coloured based on the classes such that nodes present in the same class had the same colour while grey nodes represented the non-matched metabolites. The fragmentation spectra of all the putatively annotated metabolites matched to the GNPS spectral libraries were manually validated using the metabolite annotation workflow described below.

### 3.5. Metabolite Annotation and Biological Interpretation

Metabolite features were annotated based on the following criteria: (i) molecular formula (MF) from full-scan accurate mass data, filtered through heuristic rules such as mass differences, nitrogen rules, restrictions of element numbers, isotopic fit and rings-and-double-bond equivalent; (ii) the calculated, filtered elemental composition predictions were searched against bioinformatics tools or databases such as PlantCyc (https://www.plantcyc.org/, accessed on 15 March 2021), Dictionary of Natural Product (DNP) (http://dnp.chemnetbase.com/faces/chemical/ChemicalSearch.xhtml, accessed on 15 March 2021), Chemspider (http://www.chemspider.com/, accessed on 15 March 2021), and Kyoto Encyclopedia of Genes and Genomes (KEGG) (https://www.genome.jp/kegg/, accessed 15 on March 2021), to putatively assign compound names to the MF; (iii) structural elucidation was performed based on the fragmentation patterns by examining the MS^1^ and MS^E^ spectra of the metabolites; and (iv) putative annotations of metabolites were also compared to the available literature, considering their respective chromatographic elution profiles on a reverse-phase column. In the current study, metabolites were putatively annotated to level 2 of the Metabolomics Standards Initiative (MSI) [55].

All annotated and targeted metabolites (Tables S2 and S4) were used for metabolic pathway and network analyses. Metabolic pathway analysis was performed with the Metabolomics Pathway Analysis (MetPA) component of the MetaboAnalyst bioinformatics tool suite (version 5.0). This enabled the identification of the affected metabolic pathways, analysis thereof, and visualization. MetPA uses high-quality KEGG metabolic pathways as the backend knowledge base. In addition to the existing literature, the use of these bioinformatics tools (for pathway analysis) provided a framework to partially map the molecular landscape of the metabolism under study, enabling the biological interpretability of observed changes in a metabolome view [44]. To globally visualize the metabolite changes, a correlation network was computed using MetaMapp (http://metamapp.fiehnlab.ucdavis.edu/, accessed on 30 April 2021). MetaMapp-encoded chemical structures of all the identified metabolites were retrieved from the PubChem and KEGG databases, and the *p*-values and fold changes were obtained from OPLS-DA-derived descriptive statistics (Table S3). A Tanimoto score threshold of 0.7 was used to define the similarity cut-off among metabolites. The generated networks were visualized using Cytoscape v3.8.1 [56].

## 4. Conclusions

Understanding the modes of action involved in biostimulant-mediated growth promotion and stress resilience is one of the critical steps necessary for the full implementation and integration of biostimulants into agricultural practices. Thus, this present study intended to decode a metabolic choreography that defines the effects of an HS-based biostimulant on maize plants, under normal and starved conditions, in a greenhouse setting. Although further investigation may be needed to build on our findings, the model derived from this metabolomics study suggests that the HS-biostimulant induced a metabolic reprogramming in maize plants towards growth promotion and the alleviation of starvation stress. Molecular networking approaches aided in characterizing the HS-altered chemical space. In more detail, a wide and coordinated range of metabolic processes was involved in the response of maize plants to HS treatments. Impacted metabolic pathways included amino acid metabolism, phenylalanine metabolism, and alpha-linolenic acid metabolism, among others, involving a spectrum of metabolite classes such as amino acids, phytohormones, lipids, HCA compounds and flavonoids which are involved in growth promotion and nutrient stress alleviation. Furthermore, metabolic network analysis revealed some qualitative characteristics of HS effects on maize metabolism under nutrient starvation: a complex structural interconnectivity between altered metabolites involved in stress alleviation and metabolite hubs depicting possible biochemical regulatory mechanisms, which can be investigated further. These HS-induced multilayered metabolic reconfigurations in maize plants could generally be linked to morphophysiological data such as chlorophyll content, nutrient assimilation, and changes in biomass. The knowledge generated from this work provides a morphophysiological and metabolomic gateway to the mechanisms underlying the effects of HS-biostimulant on plants. Such insights lay a foundation for advancement of the biostimulant industry and incorporation of these formulations in agronomic practices, for sustainable food security.

## Figures and Tables

**Table 1:** Humic substance application rates (L/ha) and nutrient solution (%) applied to the plant sample groups.

Treatment	HS Application Rate (L/ha)	Nutrient Solution (%)
Control 1 (C1, Starved)	0	40
Starved + HS	20	40
Control 2 (C2, non-starved)	0	100
Non-starved + HS	20	100

## Data Availability

The study design information, LC-MS raw data, analyses and data processing information, and the meta-data will be deposited in the EMBL-EBI metabolomics repository—MetaboLights (Haug et al., 2020), with the identifier MTBLS2854 (https://www.ebi.ac.uk/metabolights/MTBLS2854).

## Supplementary Materials

The following are available online at https://www.mdpi.com/article/10.3390/metabo11060403/s1, the GNPS links; Figure S1: Morphophysiological assessments; Figure S2: Relative quantification and chemometric models; Table S1: Metabolic pathway analysis; Table S2: Annotated metabolites; Table S3: MetaMapp data; Table S4: MRM-MS method details.
