# Supplementary material for: A Metabolic Choreography of Maize Plants Treated with a Humic Substance-Based Biostimulant under Normal and Starved Conditions
Source: Metabolites. 2021 Jun 20;11(6):403. doi: 10.3390/metabo11060403 (PMC8235525; doi:10.3390/metabo11060403)
Supplement: Supplementary file 1 [file metabolites-11-00403-s001.zip › metabolites-1251463-supplementary.pdf]

Supplementary Materials

GNPS LINK DIRECTORIES

GNPS JOB links:

(1) Negative mode:

FBMN: https://gnps.ucsd.edu/ProteoSAFe/status.jsp?task=2d4d953a6c0846da81b4f804a7515a28

MolNetEnhancer:
https://gnps.ucsd.edu/ProteoSAFe/status.jsp?task=af3336a79c90441e9d990e8d55066302

(2) Positive mode:

FBMN: https://gnps.ucsd.edu/ProteoSAFe/status.jsp?task=de0059c12a2d4e9a9dec044eb60813e9

MolNetEnhancer:
https://gnps.ucsd.edu/ProteoSAFe/status.jsp?task=a8af348c4de74d18811b3af8ee041382

Spectral information of caffeoylquinic acid (m/z 353.08)

https://metabolomics-usi.ucsd.edu/mirror?usi1=mzspec%3AGNPS%3ATASK-2d4d953a6c0846da81b4f804a7515a28-spectra%2Fspecs_ms.mgf%3Ascan%3A2381&usi2=mzspec%3AGNPS%3AGNPS%3AGNPS-LIBRARY%3Aaccession%3ACCMSLIB00005743945&width=10.0&height=6.0&mz_min=&mz_max=&max_intensity=150.0&grid=true&annotate_peaks=[[191.0522,353.0895],[135.0446,191.0557,353.0876]]&annotate_precision=4&annotation_rotation=90&cosine=standard&fragment_mz_tolerance=0.02

Table S1: Metabolic pathway analysis (MetPA).

**Arranged by pathway impact**					
**Number**	**Pathway name**	**Total**	**Hits**	**p-value**	**Impact**
**1**	Isoquinoline alkaloid biosynthesis	6	1	0.13	0.50
**2**	Phenylalanine metabolism	11	1	0.23	0.47
**3**	alpha-Linolenic acid metabolism	28	3	0.02	0.38
**4**	Tryptophan metabolism	28	2	0.13	0.32
**5**	Glycine, serine and threonine metabolism	33	4	0.01	0.30
**6**	Cysteine and methionine metabolism	46	5	0.00	0.20
**8**	Stilbenoid, diarylheptanoid and gingerol biosynthesis	8	1	0.17	0.13
**9**	Alanine, aspartate and glutamate metabolism	22	2	0.09	0.13
**10**	Glyoxylate and dicarboxylate metabolism	29	2	0.14	0.12
**7**	Aminoacyl-tRNA biosynthesis	46	12	0.00	0.11
**11**	Tyrosine metabolism	16	1	0.31	0.11
**12**	Arginine and proline metabolism	34	1	0.55	0.07
**13**	Sulfur metabolism	15	2	0.04	0.06
**14**	Citrate cycle (TCA cycle)	20	1	0.37	0.05
**15**	Phenylpropanoid biosynthesis	46	2	0.28	0.03
**16**	Phenylalanine, tyrosine and tryptophan biosynthesis	22	3	0.01	0.02
**17**	Flavonoid biosynthesis	47	1	0.67	0.02
**18**	Glutathione metabolism	26	1	0.46	0.01
**19**	Carotenoid biosynthesis	43	1	0.64	0.01
**20**	Purine metabolism	63	1	0.78	0.00
**Arranged by p-value**					
**7**	Aminoacyl-tRNA biosynthesis	46	12	0.00	0.11
**6**	Cysteine and methionine metabolism	46	5	0.00	0.20

**21**	Cyanoamino acid metabolism	29	4	0.00	-
**5**	Glycine, serine and threonine metabolism	33	4	0.01	0.30
**16**	Phenylalanine, tyrosine and tryptophan biosynthesis	22	3	0.01	0.02
**22**	Valine, leucine and isoleucine biosynthesis	22	3	0.01	-
**23**	Glucosinolate biosynthesis	65	5	0.01	-
**3**	alpha-Linolenic acid metabolism	28	3	0.02	0.38
**24**	Sulfur metabolism	15	2	0.04	0.06
**25**	Carbon fixation in photosynthetic organisms	21	2	0.08	-

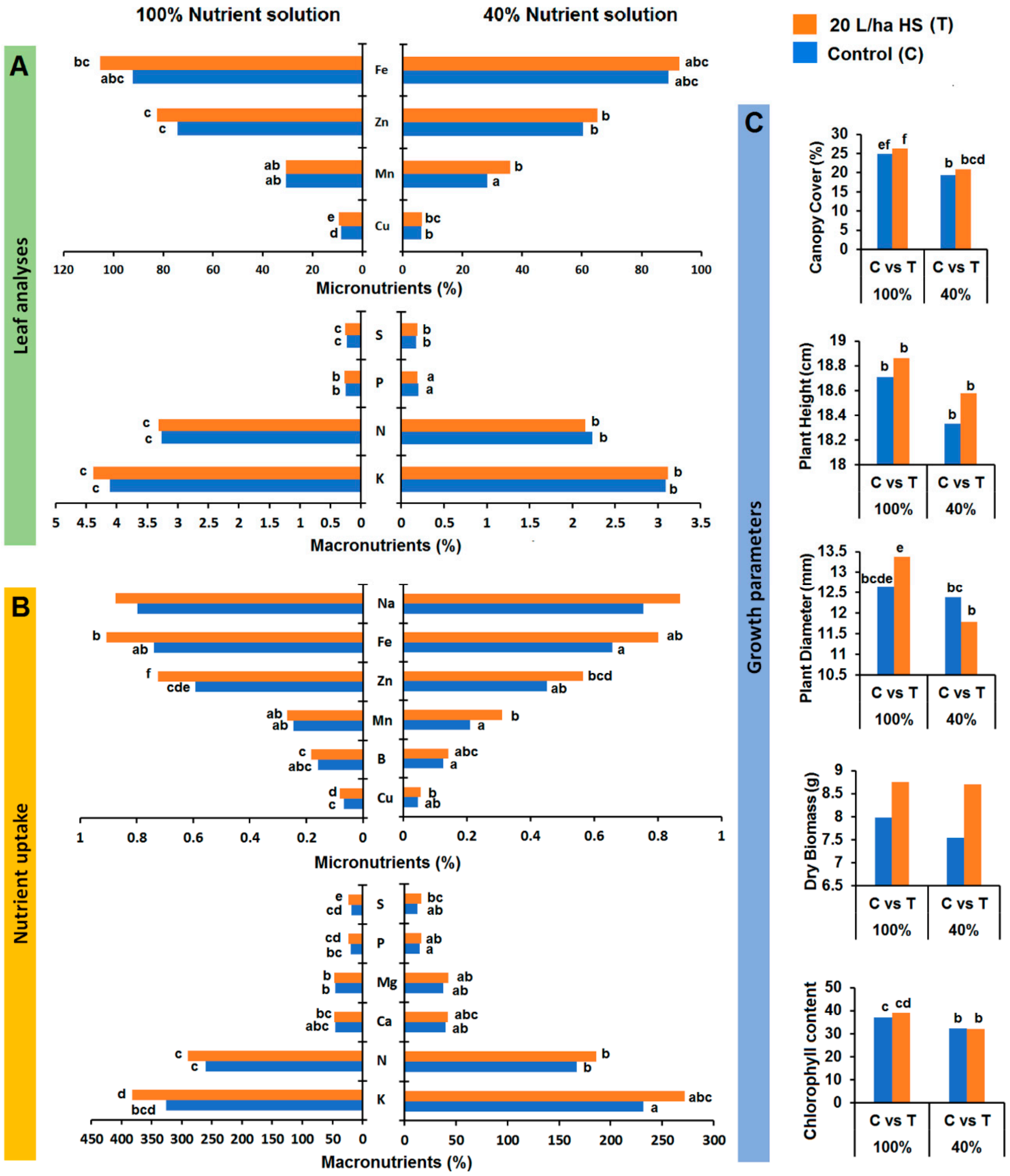

Figure S1: Morphophysiological assessments. (A-B) The humic substances (HS) application improved the nutrient uptake and leaf content under starved (40% nutrition) and non-starved conditions (100% nutrition). (C) The growth parameters were also enhanced by HS treatment under starved and non-starved conditions. Treatments containing the same letters are not significantly different from one another ($p \leq 0.05$), and different letters indicate statistically significant differences between treatments; $p \leq 0.05$.

Table S2: Annotated metabolites (level 2, MSI)

Metabolite name	Abbreviations	Rt (min)	m/z	Fragment ions	Adduct	Molecular Formula	Class
Isocitric acid	Iso	1.24	191.04	173,129,111,85	$[M-H]^-$	$C_6H_9O_7$	Organic acid
Adenosine	Ade	1.67	268.09	136	$[M+H]^+$	$C_{10}H_{13}N_5O_4$	Nucleotide
Isoleucine	Iso	1.92	132.00	86,69	$[M+H]^+$	$C_6H_{13}NO_2$	Amino acids
Caffeoylglucarate 1	Cafglu 1	2.02	371.06	209,173,143,129,85	$[M-H]^-$	$C_{15}H_{17}O_{11}$	HCA compounds
Galloyl-hexoside (Glucogallin)	Gal-hex/galloyl-hex	2.35	331.06	168,125	$[M-H]^-$	$C_{13}H_{16}O_{10}$	HCA compounds
Caffeoylglucarate 2	Cafglu 2	2.45	371.06	209,173,143,129,85	$[M-H]^-$	$C_{15}H_{17}O_{11}$	HCA compounds
Caffeoylglucarate 3	Cafglu 3	2.65	371.06	209,173,143,129,85	$[M-H]^-$	$C_{15}H_{17}O_{11}$	HCA compounds
3-Caffeoylquinic acid	3-CafQA	3.22	353.91	191, 179	$[M-H]^-$	$C_{16}H_{18}O_9$	HCA compounds
Caffeoylhydroxycitric acid 1	Caf-hyd 1	3.39	369.04	207, 189, 127	$[M-H]^-$	$C_{15}H_{14}O_{11}$	HCA compounds
trans-3-Caffeoyquinic acid	3-CafQA	3.43	353.09	191, 179	$[M-H]^-$	$C_{16}H_{18}O_9$	HCA compounds
Caffeoylhydroxycitric acid 2	Caf-hyd 2	3.84	369.04	207, 189, 127	$[M-H]^-$	$C_{15}H_{14}O_{11}$	HCA compounds
Caffeoylhydroxycitric acid 3	Caf-hyd 3	4.08	369.04	207, 189, 127	$[M-H]^-$	$C_{15}H_{14}O_{11}$	HCA compounds
Coumaroylquinic acid	CouQA	4.15	337.09	163	$[M-H]^-$	$C_{16}H_{18}O_8$	HCA compounds
Kynurenic acid	Kyn	4.15	190.04	172,162,144	$[M+H]^+$	$C_{10}H_7NO_3$	Amino acids
5−Caffeoylquinic acid	5-CafQA	4.40	353.08	191	$[M-H]^-$	$C_{16}H_{18}O_9$	HCA compounds
3-Feruloylquinic acid 1	3-FerQA	4.49	367.95	193, 134	$[M-H]^-$	$C_{17}H_{20}O_9$	HCA compounds
Coumaroylhydroxycitric acid	Co-hyd	4.67	353.07	189, 127	$[M-H]^-$	$C_{15}H_{14}O_{10}$	HCA compounds
3-Feruloylquinic acid 2	3-FerQA 2	4.72	367.10	193, 134	$[M-H]^-$	$C_{17}H_{20}O_9$	HCA compounds
Luteolin diglucoside	Lut-diglu	4.88	609.14	447,383,353,327,285,267,188	$[M-H]^-$	$C_{27}H_{30}O_{16}$	Flavonoids
Feruloylhydroxycitric acid 1	Fer-hyd 1	5.02	383.06	188, 127	$[M-H]^-$	$C_{16}H_{16}O_{11}$	HCA compounds
4-Caffeoylquinic acid	4-CafQA	5.08	353.05	191, 173	$[M-H]^-$	$C_{16}H_{18}O_9$	HCA compounds
Feruloylhydroxycitric acid 2	Fer-hyd 2	5.25	383.06	188, 127	$[M-H]^-$	$C_{16}H_{16}O_{11}$	HCA compounds
Kaempferol neohesperidoside	Kae-neo	5.63	593.15	473, 447, 327, 285	$[M-H]^-$	$C_{27}H_{30}O_{15}$	Flavonoids
beta-glucosyl (sinapoylglucosyl)isovitexin	Isovitexin der	5.65	962.28	593,563,447,367,223	$[M-H]^-$	$C_{44}H_{50}O_{24}$	Flavonoids
Luteolin rutinoside	Lut-rut	5.74	593.15	447, 285	$[M-H]^-$	$C_{27}H_{30}O_{15}$	Flavonoids
Kaempferol rutinoside rhamnoside	Kae-rut-rha	5.98	739.21	284	$[M-H]^-$	$C_{33}H_{40}O_{19}$	Flavonoids
Tricin diglucuronoside	Tri-diglucu	6.19	681.13	351,300,193	$[M-H]^-$	$C_{29}H_{30}O_{19}$	Flavonoids

Quercetin rhamnoside glucoside	Que-rha-glu	6.22	609.15	351,300,178	$[M-H]^{-}$	$C_{27}H_{30}O_{16}$	Flavonoids
Kaempferol rutinoside	Kae-rut	6.31	593.15	324,284	$[M-H]^{-}$	$C_{27}H_{30}O_{15}$	Flavonoids
Rutin	Rut	6.40	609.15	301, 300, 271	$[M-H]^{-}$	$C_{27}H_{30}O_{16}$	Flavonoids
Dillenetin glucoside glucuronide	Dil-glu-glucu	6.41	667.15	329	$[M-H]^{-}$	$C_{29}H_{32}O_{18}$	Flavonoids
Malvidin glucoside	Mal-glu	6.70	493.13	331	$[M+H]^{+}$	$C_{23}H_{25}O_{12}^{+}$	Flavonoids
Corchoionoside	Cor	6.82	399.16	219	$[M-H]^{-}$	$C_{19}H_{28}O_{9}$	Flavonoids
Isorhamnetin rutinoside	Iso-rut	6.84	623.16	315	$[M-H]^{-}$	$C_{28}H_{32}O_{16}$	Flavonoids
Kaemperol rhamnosyl glucoside	Kae-rha-glu	6.87	593.15	285	$[M-H]^{-}$	$C_{27}H_{30}O_{15}$	Flavonoids
Luteolin diglucuronide	Lut-diglucu	6.94	637.14		$[M-H]^{-}$	$C_{27}H_{26}O_{18}$	Flavonoids
Kaempferitrin	Kaempferitrin	7.01	577.15	473,437,413,285	$[M-H]^{-}$	$C_{27}H_{30}O_{14}$	Flavonoids
Tricin (erythro-beta-guaiacylglyceryl) ether glucopyranoside	Tricin der 1	7.04	687.19	329	$[M-H]^{-}$	$C_{33}H_{36}O_{16}$	Flavonoids
Maysin	Maysin	7.19	575.14	411, 473,337, 285, 189	$[M-H]^{-}$	$C_{27}H_{28}O_{14}$	Flavonoids
Luteolin neohesperidoside	Lut-neo	7.34	593.15	575,473,285	$[M-H]^{-}$	$C_{27}H_{30}O_{15}$	Flavonoids
Tricin 4'-O-(threo-beta-guaiacylglyceryl) ether	Tricin der 2	9.64	525.13	329	$[M-H]^{-}$	$C_{27}H_{26}O_{11}$	Flavonoids
Traumatic acid	Tra/Trau	10.24	227.12	183,165	$[M-H]^{-}$	$C_{12}H_{20}O_{4}$	Lipids
Oxo-dihydroxy-octadecadienoic acid	OxoDioct	10.38	325.20	307,227,209,153	$[M-H]^{-}$	$C_{18}H_{30}O_{5}$	Lipids
Oxododecanoic acid	Oxo	10.44	213.14	209,195,183,171	$[M-H]^{-}$	$C_{12}H_{22}O_{3}$	Lipids
TriHOME	TriHOME	11.12	329.23	327,293,211,189	$[M-H]^{-}$	$C_{18}H_{34}O_{5}$	Lipids
Hydroperoxy-eicosadienoic acid	HpEDE	11.90	339.21	307,289,245	$[M-H]^{-}$	$C_{20}H_{36}O_{4}$	Lipids
Hydroperoxy-octadecatrienoic acid 1	HpOTrE 1	12.42	309.20	291	$[M-H]^{-}$	$C_{18}H_{30}O_{4}$	Lipids
Dihydroxy-octadecadienoic acid	DiHODE	12.60	311.22	309,293,291,211,201	$[M-H]^{-}$	$C_{18}H_{32}O_{4}$	Lipids
Dihydroxy-octadecatrienoate	Dota	12.80	309.20	197,171	$[M-H]^{-}$	$C_{18}H_{30}O_{4}$	Lipids
Hydroperoxy-octadecatrienoic acid	HpOTrE 2	12.98	309.20	291,263,197	$[M-H]^{-}$	$C_{18}H_{30}O_{4}$	Lipids
DGMG 18:3 Gingerglycolipid A 1	DGMG 18:3	13.29	721.37	675,397,277	$[M+HCOO]^{-}$	$C_{33}H_{56}O_{14}$	Lipids
DGMG 18:3/ Gingerglycolipid A 2	DGMG 18:3	13.62	721.37	675,397,277	$[M+HCOO]^{-}$	$C_{33}H_{56}O_{14}$	Lipids
Oxo-(pentenyl)cyclopentaneoctanoic acid	OPC	14.37	293.21	279, 233	$[M-H]^{-}$	$C_{18}H_{30}O_{3}$	Lipids
MGMG 18:3 1	MGMG 18:3	14.53	559.31	515,277,253	$[M+HCOO]^{-}$	$C_{27}H_{46}O_{9}$	Lipids
Monolinolein	Lino-Gly 1	14.60	353.26	335,261,243,109	$[M+H]^{+}$	$C_{21}H_{38}O_{4}$	Lipids
MGMG 18:3 2	MGMG 18:3	14.83	559.31	515,277,253	$[M+HCOO]^{-}$	$C_{27}H_{46}O_{9}$	Lipids

Linoleoyl Glycerol	Lino-Gly 2	14.89	353.26	335,261,243,109	$[M+H]^+$	$C_{21}H_{38}O_4$	Lipids
OPDA	OPDA	14.90	291.19		$[M-H]^-$	$C_{18}H_{28}O_3$	Lipids

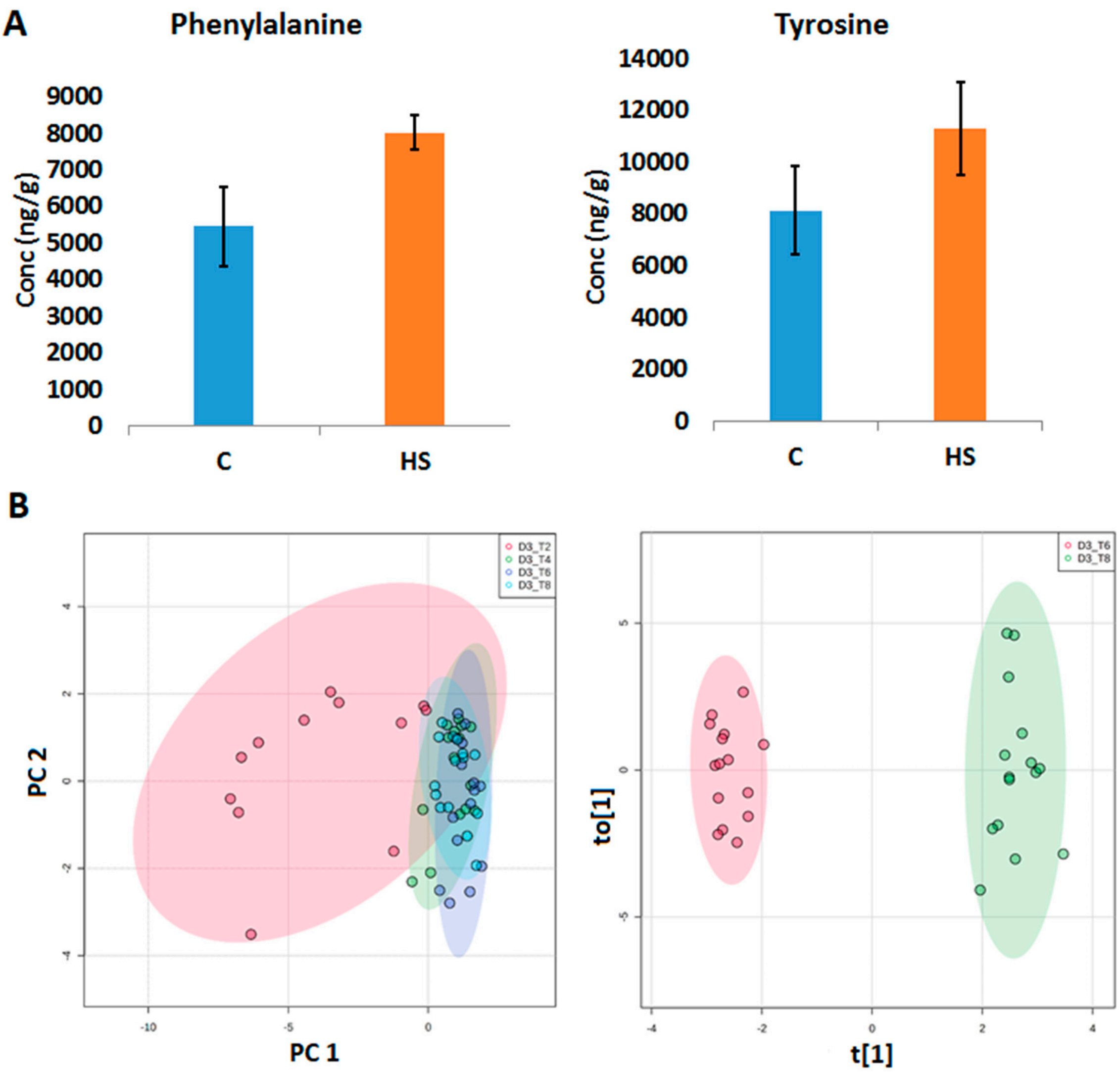

Figure S2: Relative quantification and chemometric models. (**A**) Absolute quantification of aromatic amino acids. (**B**) Typical chemometrics modeling, applied in this study: (i) principal component analysis (PCA), a score plot (PC1 *vs* PC2) of a 5-component model (of the Pareto-scaled data matrix X), with cumulative R^2 of 53.2% (explained variation). The unsupervised modelling (PCA) provided a global overview of the data as infographically shown in the PCA scores plots (an example shown in this Figure), allowing the identification of sample grouping and natural clustering in multivariate space: the starved samples (red) group away from the other treatments, whereas the HS-treated samples overlap (cluster together) with samples of normal conditions (control 2, **Table 1**). (ii) A typical OPLS-DA modelling, in this case, a score plot of an OPLS-DA model separating 'control (red) *vs*. HS-treated plants (green)' (under normal conditions), with 1 + 2 + 0 components, $R^2X = 0.611$, $Q^2 = 0.915$, CV-ANOVA p-value less than 0.05. Evaluation of this multivariate (binary) classifier (OPLS-DA) helps in extracting the metabolite variables underlying the classification (separation) of groups.

Table S3: Metamapp data

				HS effects under non-starved conditions		Effects of starvation		HS effects under starvation	
PubChem_ID	KEGG_ID	SMILES	Compound_Name	p-value	Fold change	p-value	Fold-change	p-value	Fold-change
535	C01234	C1CC1(C(=O)O)N	1-Aminocyclopropanecarboxylic acid	2.98E-02	3.18	2.33E-01	1.34	5.18E-01	1.47
10256	C08493	C1=CC=C2C(=C1)C(=CN2)C=O	1H-Indole-3-carboxaldehyde	8.59E-01	1.10	1.51E-01	0.76	6.77E-01	1.04
12310831		C1[C@H]([C@@H]([C@@H](C[C@]1(C(=O)O)O)OC(=O)/C=C\C2=CC(=C(C=C2)O)O)O)O	3-Caffeoylquinic acid	6.30E-02	0.28	2.09E-02	0.73	5.79E-01	0.55
10133609	C02572	COC1=C(C=CC(=C1)C=CC(=O)OC2CC(CC(C2O)O)(C(=O)O)O)O	3-Feruloylquinic acid	6.62E-01	1.32	4.44E-03	0	7.24E-02	4 352.27
58427569		C1C(C(C(CC1(C(=O)O)O)O)OC(=O)C=CC2=CC(=C(C=C2)O)O)O	4-Caffeoylquinic acid	1.37E-01	1.00	2.46E-02	0	1.34E-07	0
1794427	C00852	C1C(C(C(CC1(C(=O)O)O)OC(=O)C=CC2=CC(=C(C=C2)O)O)O)O	5-Caffeoylquinic acid	1.61E-07	0.69	5.83E-08	1.50	1.71E-01	1.21
643732	C06082	CC1=CC(=O)CC(C1(C=CC(=CC(=O)O)C)O)(C)C	Abscisic acid	1.32E-01	2.69	5.79E-04	0.67	4.65E-01	0.83
60961	C00212	C1=NC(=C2C(=N1)N(C=N2)[C@H]3[C@@H]([C@@H]([C@H](O3)CO)O)O)N	Adenosine	9.91E-01	1.00	6.47E-02	0.57	3.08E-03	0.69
5950	C00041	CC(C(=O)O)N	Alanine	1.78E-06	1.30	8.32E-02	1.18	7.40E-03	1.50
5960	C00049	C(C(C(=O)O)N)C(=O)O	Aspartic acid	2.56E-03	1.47	6.24E-08	0.63	2.50E-02	1.42
72193662		COC1=CC(=CC(=C1O)OC)C=CC(=O)OCC2C(C(C(C(O2)OC3=C(C(=C4C(=C3)OC(=CC4=O)C5=CC=C(C=C5)OC6C(C(C(C(O6)CO)O)O)O)O)C7C(C(C(C(O7)CO)O)O)O)O)O)O	beta-glucosyl (sinapoylglucosyl)isovitexin	2.28E-03	2.80	1.12E-11	0.22	5.62E-01	0.86
5459979	C03062	C1=CC(=C(C=C1C=CC(=O)OC(C(C(C(C(=O)O)O)O)O)C(=O)O)O)O	Caffeoylglucarate	5.06E-01	0.92	1.48E-03	3.75	1.66E-05	0
24797519		C1=CC(=C(C=C1C=CC(=O)OC(C(=O)O)C(CC(=O)O)(C(=O)O)O)O)O	Caffeoylhydroxycitric acid	1.04E-05	0.67	1.66E-05	1.50	4.29E-02	1.63
131751110		CC1=CC(=O)CC(C1(C=CC(=O)COC2C(C(C(C(O2)CO)O)O)O)O)(C)C	Corchoionoside	1.29E-03	0.73	1.73E-01	1.52	8.87E-02	0.81
131752858		C1=CC(=CC=C1C=CC(=O)OC(C(=O)O)C(CC(=O)O)(C(=O)O)O)O	Coumaroylhydroxycitric acid	3.22E-03	3.52	2.61E-05	1.67	1.89E-01	0.28
5281766	C10441	C1C(C(C(CC1(C(=O)O)O)O)OC(=O)C=CC2=CC=C(C=C2)O)O	Coumaroylquinic acid	1.51E-01	1.37	1.22E-01	6.27	5.30E-01	1.13
5862	C00097	C(C(C(=O)O)N)S	Cysteine	1.98E-01	1.26	2.01E-02	1.48	1.05E-01	0.75
131753022		O=C(OCC(O)COC2OC(COC1OC(CO)C(O)C(O)C1(O))C(O)C(O)C2(O))CCCCCCCC=CCC=CCC=CCC	DGMG 18:3/ Gingerglycolipid A	9.07E-01	1.02	1.37E-02	1.54	7.68E-02	1.33
52921872	C07354	CCC=CCC(C(C=CC(=O)CCCCCCCC(=O)O)O)O	Dihydroxy-octadecadienoic acid (DiHODE)	5.47E-01	1.29	1.03E-01	0.91	1.87E-03	1.78
6439106		CCCCCC=CC(C(C=CC=CCCCCCC(=O)O)O)O	Dihydroxy-octadecatrienoate (Dota)	3.79E-02	1.50	1.07E-02	2.38	2.43E-01	1.26

44259417		COC1=C(C=C(C=C1)C2=C(C(=O)C3=C(O2)C=C(C=C3OC4C(C(C(C(O4)CO)O)O)O)OC5C(C(C(C(O5)C(=O)O)O)O)O)O)OC	Dillenetin glucoside glucuronide	8.92E-01	0.98	3.93E-01	0.87	1.67E-01	1.23
131752859		COC1=C(C=CC(=C1)C=CC(=O)OC(C(=O)O)C(CC(=O)O)(C(=O)O)O)O	Feruloylhydroxycitric acid	1.72E-11	1.57	9.33E-04	0.59	6.72E-02	1.38
124021	C01158	C1=C(C=C(C(=C1O)O)O)C(=O)OC2C(C(C(C(O2)CO)O)O)O	Galloyl-hexoside	6.88E-02	0.78	2.43E-03	26.39	1.90E-01	1.11
5282869		CCCCCC(C=CC=CCCCCCCCCCC(=O)O)OO	Hydroperoxy-eicosadienoic acid (HpEDE)	3.50E-01	0.87	1.21E-01	0.86	2.73E-01	0.80
53792623	C04785	CCCCCCCCCC(CC=CC=CC=CC(=O)O)OO	Hydroperoxy-octadecatrienoic acid (HpOTrE)	3.79E-02	1.50	3.29E-01	0.84	5.98E-01	1.10
69867	C19837	C1=CC=C2C(=C1)C(=CN2)C(=O)O	Indole-3-carboxylic acid	6.43E-01	1.68	3.93E-01	0.87	9.00E-01	0.97
802	C00954	C1=CC=C2C(=C1)C(=CN2)CC(=O)O	Indoleacetic acid	4.77E-01	1.36	3.27E-01	0.83	9.00E-01	0.65
1198	C00311	C(C(C(C(=O)O)O)C(=O)O)C(=O)O	Isocitric acid	5.08E-01	0.89	4.29E-01	0.41	8.55E-01	0.95
6306	C00407	CC[C@H](C)[C@@H](C(=O)O)N	Isoleucine	3.16E-07	4.25	1.84E-01	1.47	3.87E-01	1.70
133562525	C10084	CC1C(C(C(C(O1)OCC2C(C(C(C(O2)OC3=C(OC4=CC(=CC(=C4C3=O)O)O)C5=CC(=C(C=C5)O)OC)O)O)O)O)O)O	Isorhamnetin rutinoside	3.06E-03	0.71	3.00E-01	0.56	7.76E-01	0.96
5486199	C16981	CC1C(C(C(C(O1)OC2=CC(=C3C(=C2)OC(=C(C3=O)OC4C(C(C(C(O4)C)O)O)O)C5=CC=C(C=C5)O)O)O)O)O	Kaempferitrin	6.19E-01	0.92	8.65E-01	0.98	4.27E-03	1.47
90795788	C21854	CC1C(C(C(C(O1)OC2=C(OC3=CC(=CC(=C3C2=O)O)OC4C(C(C(C(O4)CO)O)O)O)C5=CC=C(C=C5)O)O)O)O	Kaempferol 3-O-rhamnoside-7-O-glucoside	2.35E-05	0.60	6.46E-04	0.63	2.66E-06	0
5318761		CC1C(C(C(C(O1)OC2C(C(C(OC2OC3=C(OC4=CC(=CC(=C4C3=O)O)O)C5=CC=C(C=C5)O)CO)O)O)O)O)O	Kaempferol neohesperidoside	6.05E-05	0.63	2.66E-06	1.50	7.92E-04	1.65
122173234	C21833	CC1C(C(C(C(O1)OCC2C(C(C(C(O2)OC3=C(OC4=CC(=CC(=C4C3=O)O)O)C5=CC=C(C=C5)O)O)O)O)O)O)O	Kaempferol rutinoside	1.23E-06	0.69	2.80E-01	0.85	4.62E-06	4.04
44258840		CC1C(C(C(C(O1)OCC2C(C(C(C(O2)OC3=C(OC4=CC(=CC(=C4C3=O)O)OC5C(C(C(C(O5)C)O)O)O)C6=CC=C(C=C6)O)O)O)O)O)O)O	Kaempferol rutinoside rhamnoside	1.30E-01	0.91	4.09E-08	0.22	1.96E-02	1.24
3845		C1=CC=C2C(=C1)C(=O)C=C(N2)C(=O)O	Kynurenic acid	5.93E-02	1.27	7.44E-02	0.86	4.41E-01	0.89
6256628		CCCCC/C=C/C/C=C/CCCCCCCC(=O)OCC(CO)O	Linoleoyl Glycerol	1.23E-03	0.67	3.27E-03	1.51	1.92E-01	0.82
442615	C10102	C1=CC(=C(C=C1C2=CC(=O)C3=C(C(=C(C(=C3O2)[C@H]4[C@@H]([C@H]([C@@H]([C@H](O4)CO)O)O)O)O)[C@H]5[C@@H]([C@H]([C@@H]([C@H](O5)CO)O)O)O)O)O)O	Luteolin 6,8-di-C-glucoside	4.44E-01	1.13	5.18E-02	0.51	2.79E-02	2.19
5282153	C12632	C1=CC(=C(C=C1C2=CC(=O)C3=C(C=C(C=C3O2)OC4C(C(C(C(O4)C(=O)O)O)O)OC5C(C(C(C(O5)C(=O)O)O)O)O)O)O)O	Luteolin 7-O-beta-D-diglucuronide	2.27E-01	0.87	6.46E-04	0.63	2.65E-01	1.17

44258083	C12630	CC1C(C(C(C(O1)OC2C(C(C(OC2OC3=CC(=C4C(=C3)OC(=CC4=O)C5=CC(=C(C=C5)O)O)O)CO)O)O)O)O)O	Luteolin neohesperidoside	7.66E-02	0.86	3.32E-01	0.93	7.50E-01	1.02
12315422		CC1C(C(C(C(O1)OCC2C(C(C(C(O2)OC3=CC(=C4C(=C3)OC(=CC4=O)C5=CC(=C(C=C5)O)O)O)O)O)O)O)O)O	Luteolin rutinoside	7.72E-01	1.41	4.80E-04	1.50	4.80E-04	-
443652	C12140	COC1=CC(=CC(=C1O)OC)C2=[O+]C3=CC(=CC(=C3C=C2O[C@H]4[C@@H]([C@H]([C@@H]([C@H](O4)CO)O)O)O)O)O	Malvidin-3-glucoside	6.54E-07	4.22	3.18E-01	119.08	4.54E-01	1.66
70698181		C[C@@H]1[C@H]([C@@H]([C@@H]([C@H](O1)O[C@H]2[C@@H](C(=O)[C@@H](O[C@@H]2C3=C(C4=C(C=C3O)OC(=CC4=O)C5=CC(=C(C=C5)O)O)O)C)O)O)O)O	Maysin	5.90E-03	0.73	4.05E-01	6.49	1.29E-01	1.16
6137	C00073	CSCCC(C(=O)O)N	Methionine	1.03E-01	1.31	6.79E-03	0.45	9.58E-02	0.88
72984398		O=C(OCC(O)COC1OC(CO)C(O)C(O)C1(O))CCCCCCCC=CCC=CCC=CCC	MGMG 18:3	4.08E-03	0.42	1.26E-02	0.43	3.75E-01	1.33
5283469		CCCCC/C=C\C/C=C\CCCCCCCC(=O)OCC(CO)O	Monolinolein	1.93E-03	0.52	1.69E-01	0.64	4.09E-01	1.30
4448	C01226	CCC=CCC1C(C=CC1=O)CCCCCCCC(=O)O	OPDA	1.59E-01	0.26	1.74E-01	0.47	5.83E-01	1.40
440477	C04780	CCC=CCC1C(CCC1=O)CCCCCCCC(=O)O	Oxo-(pentenyl)cyclopentaneoctanoic acid	2.04E-04	0.77	5.08E-10	0.50	1.18E-02	1.28
25755602	C20186	CCCCCC=CCC=CC(CCC(CCCC(=O)O)O)O	Oxo-dihydroxy-octadecadienoic acid	1.08E-03	0.69	8.26E-01	1.04	5.16E-02	1.36
439717	C02367	C(CCCCCC(=O)O)CCCCC=O	Oxododecanoic acid	1.08E-03	0.69	1.08E-03	0.69	1.99E-03	1.41
6140	C00079	C1=CC=C(C=C1)CC(C(=O)O)N	Phenylalanine	8.31E-06	1.47	3.44E-02	0.63	1.37E-02	1.89
145742	C00148	C1CC(NC1)C(=O)O	Proline	6.64E-01	1.09	3.61E-06	0.44	8.87E-04	1.31
6325870	C19796	C[C@H]1[C@@H]([C@H]([C@H]([C@@H](O1)OC2=C(OC3=CC(=CC(=C3C2=O)O)O[C@H]4[C@@H]([C@H]([C@@H]([C@H](O4)CO)O)O)O)C5=CC(=C(C=C5)O)O)O)O)O	Quercetin 3-O-rhamnoside 7-O-glucoside	2.53E-04	0.55	5.68E-01	1.10	7.72E-01	0.95
5280805	C05625	CC1C(C(C(C(O1)OCC2C(C(C(C(O2)OC3=C(OC4=CC(=CC(=C4C3=O)O)O)C5=CC(=C(C=C5)O)O)O)O)O)O)O)O	Rutin	2.53E-04	0.55	8.16E-03	1.50	8.22E-03	0.00
338	C00805	C1=CC=C(C(=C1)C(=O)O)O	Salicylic acid	1.78E-06	1.82	9.00E-01	0.75	9.00E-01	0.90
5951	C00065	C(C(C(=O)O)N)O	Serine	8.00E-01	1.00	1.07E-07	0.23	5.65E-01	1.05
6288	C00188	CC(C(C(=O)O)N)O	Threonine	5.91E-06	1.48	3.96E-04	0.65	7.46E-03	1.30
6443509	C16308	C(CCO)CC(CCC=CC=CC(=O)O)O	TRAUMATIC ACID	1.69E-02	1.71	1.09E-01	1.38	3.15E-01	1.20
122376003		COC1=CC(=CC(=C1OC(CO)C(C2=CC(=C(C=C2)O)OC)OC3C(C(C(C(O3)CO)O)O)O)OC)C4=CC(=O)C5=C(C=C(C=C5O4)O)O	Tricin (erythro-beta-guaiacylglyceryl) ether glucopyranoside	5.72E-01	0.93	3.36E-03	0.54	5.72E-01	1.14
53262853	C10193	COC1=CC(=CC(=C1OC(CO)C(C2=CC(=C(C=C2)O)OC)O)OC)C3=CC(=O)C4=C(C=C(C=C4O3)O)O	Tricin 4'-O-(threo-beta-guaiacylglyceryl) ether	2.99E-01	0.82	3.53E-02	0.64	4.29E-01	1.19

131752191		COC1=CC(=CC(=C1O)OC)C2=CC(=O)C3=C(C=C(C=C3O2)OC4C(C(C(C(O4)C(=O)O)O)O)OC5C(C(C(C(O5)C(=O)O)O)O)O)O	Tricin diglucuronoside	3.13E-01	1.11	3.61E-02	0.78	3.82E-01	1.13
9858729	C14833	CCCCCC(C(C=CC(CCCCCCCC(=O)O)O)O)O	TriHOME	1.39E-02	1.38	2.50E-01	0.81	3.64E-02	1.49
6305	C00078	C1=CC=C2C(=C1)C(=CN2)CC(C(=O)O)N	Tryptophan	7.90E-01	1.00	1.25E-04	0.61	5.74E-04	1.52
6057	C00082	C1=CC(=CC=C1CC(C(=O)O)N)O	Tyrosine	9.72E-04	1.42	9.00E-01	1.00	1.09E-02	1.25
6287	C00183	CC(C)C(C(=O)O)N	Valine	6.59E-06	1.66	6.18E-01	0.98	5.83E-02	1.23

Table S4. The **MRM-MS method** developed and optimized by direct infusion and the collision energy (CE) optimized for transitions of each compound using the MRM optimization method tool.

Compound name	Rt (min)	Ion mode	*m/z*	Transition	CE (eV)	Quadrupole 1 (Q1), V	Quadrupole 3 (Q3), V	Dwell time (msec)
<u>Amino acids</u>								
**Proline (Pro)**	1.503	$[M+H]^+$	116.20	116.20>70.15<br>116.20>43.10	-18.0<br>-28.0	-14.0<br>-13.0	-11.0<br>-15.0	17.0<br>17.0
**Cysteine (Cys)**	1.317	$[M+H]^+$	241.20	151.90	-14.0	-12.0	-15.0	37.0
**Serine (Ser)**	1.341	$[M+H]^+$	106.20	106.20>59.95<br>106.20>88.10	-13.0<br>-13.0	-12.0<br>-12.0	-10.0<br>-18.0	17.0<br>17.0
**Alanine (Ala)**	1.406	$[M+H]^+$	90.20	90.20>44.05<br>90.20>44.90	-13.0<br>-30.0	-10.0<br>-18.0	-15.0<br>-16.0	17.0<br>17.0
**Threonine (Thr)**	1.409	$[M+H]^+$	120.20	120.20>56.05<br>120.20>74.10	-16.0<br>-12.0	-13.0<br>-13.0	-20.0<br>-28.0	17.0<br>17.0
**Aspartic acid (Asp)**	1.410	$[M+H]^+$	134.05	134.05>74.10	-15.0	-10.0	-13.0	37.0
**Valine (Val)**	1.730	$[M+H]^+$	118.20	118.20>72.10<br>118.20> 55.05	-12.0<br>-23.0	-14.0<br>-14.0	-12.0<br>-23.0	17.0<br>17.0
**Methionine (Met)**	2.142	$[M+H]^+$	150.20	150.20>60.90<br>150.20>56.10	-17.0<br>-24.0	-10.0<br>-11.0	-20.0<br>-24.0	17.0<br>17.0
**Tyrosine (Tyr)**	3.292	$[M+H]^+$	182.00	182.0>136.10	-14.0	-13.0	-24.0	37.0
**Phenylalanine (Phe)**	5.929	$[M+H]^+$	166.00	166.00>120.10	-14.0	-12.0	-21.0	131.0

**Tryptophan (Trp)**	6.774	$[M+H]^+$	205.20	205.20>188.05	-11.0	-14.0	-19.0	64.0
				205.20>146.10	-17.0	-14.0	-14.0	64.0
**<u>Hormones</u>**								
**Abscisic acid (ABA)**	7.917	$[M+H]^+$	265.10	265.10>247.20	-8.0	-20.0	-20.0	65.6
				265.10>229.30	-10.0	-13.0	-24.0	65.6
				265.10>201.15	-13.0	-13.0	-21.0	65.6
**Indole-3-acetic acid (IAA)**	26.81	$[M+H]^+$	176.10	176.10>130.10	-15.0	-20.0	-20.0	65.6
				176.10>77.20	-43.0	-12.0	-20.0	65.6
				176.10>103.10	-30.0	-12.0	-22.0	65.6
**Salicylic acid (SA)**	22.598	$[M-H]^-$	137.00	137.00>92.95	15.0	20.0	20.0	65.6
				137.00>65.00	28.0	14.0	10.0	65.6
				137.00>75.05	32.0	14.0	27.0	65.6
**Amino-cyclopropane carboxylic acid (ACC)**	1.58	$[M+H]^+$	101.60	101.60>56.20	-14.0	-18.0	-21.0	65.6
				101.60>28.15	-23.0	-18.0	-10.0	65.6
				101.60>30.20	-37.0	-18.0	-30.0	65.6
**Indole-3-carboxaldehyde* (I3A)**	16.602	$[M+H]^+$	146.05	146.05	-25.0	-	-	100.0
**Indole-3-carboxylic acid* (I3CA)**	22.002	$[M+H]^+$	161.95	161.95	-15.0	-	-	100.0

* These compounds did not fragment, thus quantified using single ion monitoring (SIM)
